# Association of Sex With Neurobehavioral Markers of Executive Function in 2-Year-Olds at High and Low Likelihood of Autism

**DOI:** 10.1001/jamanetworkopen.2023.11543

**Published:** 2023-05-04

**Authors:** Tanya St. John, Annette M. Estes, Heather C. Hazlett, Natasha Marrus, Catherine A. Burrows, Kevin Donovan, Santiago Torres Gomez, Rebecca L. Grzadzinski, Julia Parish-Morris, Rachel Smith, Martin Styner, Dea Garic, Juhi Pandey, Chimei M. Lee, Robert T. Schultz, Kelly N. Botteron, Lonnie Zwaigenbaum, Joseph Piven, Stephen R. Dager

**Affiliations:** 1 Department of Speech and Hearing Science, University of Washington, Seattle; 2 University of Washington Autism Center, University of Washington, Seattle; 3 Carolina Institute for Developmental Disabilities, Carrboro, North Carolina; 4 Department of Psychiatry, University of North Carolina at Chapel Hill School of Medicine, Chapel Hill; 5 Department of Psychiatry, Washington University School of Medicine in St Louis, Missouri; 6 Department of Pediatrics, University of Minnesota, Minneapolis; 7 Department of Biostatistics, University of Pennsylvania, Philadelphia; 8 McGill Centre for Integrative Neuroscience, Montreal Neurological Institute, McGill University, Montreal, Quebec, Canada; 9 Center for Autism Research, Children’s Hospital of Philadelphia, University of Pennsylvania Perelman School of Medicine, Philadelphia; 10 Department of Pediatrics, University of Alberta, Edmonton, Alberta, Canada; 11 Department of Radiology, University of Washington Medical Center, Seattle

## Abstract

**Importance:**

Children with autism and their siblings exhibit executive function (EF) deficits early in development, but associations between EF and biological sex or early brain alterations in this population are largely unexplored.

**Objective:**

To investigate the interaction of sex, autism likelihood group, and structural magnetic resonance imaging alterations on EF in 2-year-old children at high familial likelihood (HL) and low familial likelihood (LL) of autism, based on having an older sibling with autism or no family history of autism in first-degree relatives.

**Design, Setting, and Participants:**

This prospective cohort study assessed 165 toddlers at HL (n = 110) and LL (n = 55) of autism at 4 university-based research centers. Data were collected from January 1, 2007, to December 31, 2013, and analyzed between August 2021 and June 2022 as part of the Infant Brain Imaging Study.

**Main Outcomes and Measures:**

Direct assessments of EF and acquired structural magnetic resonance imaging were performed to determine frontal lobe, parietal lobe, and total cerebral brain volume.

**Results:**

A total of 165 toddlers (mean [SD] age, 24.61 [0.95] months; 90 [54%] male, 137 [83%] White) at HL for autism (n = 110; 17 diagnosed with ASD) and LL for autism (n = 55) were studied. The toddlers at HL for autism scored lower than the toddlers at LL for autism on EF tests regardless of sex (mean [SE] B = −8.77 [4.21]; 95% CI, −17.09 to −0.45; η^2^_p_ = 0.03). With the exclusion of toddlers with autism, no group (HL vs LL) difference in EF was found in boys (mean [SE] difference, −7.18 [4.26]; 95% CI, 1.24-15.59), but EF was lower in HL girls than LL girls (mean [SE] difference, −9.75 [4.34]; 95% CI, −18.32 to −1.18). Brain-behavior associations were examined, controlling for overall cerebral volume and developmental level. Sex differences in EF-frontal (B [SE] = 16.51 [7.43]; 95% CI, 1.36-31.67; η^2^_p_ = 0.14) and EF-parietal (B [SE] = 17.68 [6.99]; 95% CI, 3.43-31.94; η^2^_p_ = 0.17) associations were found in the LL group but not the HL group (EF-frontal: B [SE] = −1.36 [3.87]; 95% CI, −9.07 to 6.35; η^2^_p_ = 0.00; EF-parietal: B [SE] = −2.81 [4.09]; 95% CI, −10.96 to 5.34; η^2^_p_ = 0.01). Autism likelihood group differences in EF-frontal (B [SE] = −9.93 [4.88]; 95% CI, −19.73 to −0.12; η^2^_p_ = 0.08) and EF-parietal (B [SE] = −15.44 [5.18]; 95% CI, −25.86 to −5.02; η^2^_p_ = 0.16) associations were found in girls not boys (EF-frontal: B [SE] = 6.51 [5.88]; 95% CI, −5.26 to 18.27; η^2^_p_ = 0.02; EF-parietal: B [SE] = 4.18 [5.48]; 95% CI, −6.78 to 15.15; η^2^_p_ = 0.01).

**Conclusions and Relevance:**

This cohort study of toddlers at HL and LL of autism suggests that there is an association between sex and EF and that brain-behavior associations in EF may be altered in children at HL of autism. Furthermore, EF deficits may aggregate in families, particularly in girls.

## Key Points

**Question:**

Are executive function (EF) differences early in development associated with sex, familial likelihood for autism, and alterations in brain volume?

**Findings:**

In this cohort study of 165 children aged 24 months at high and low familial likelihood for autism, girls and boys at high likelihood had significantly lower EF than girls and boys at low likelihood. Differences in brain-behavior associations across sex and familial likelihood for autism were also found.

**Meaning:**

This pattern of findings suggests that EF impairments may aggregate in families with an autistic proband, particularly in girls.

## Teaser

This cohort study investigates the association of sex, likelihood of autism based on family history, and structural brain alterations with executive function in children aged 2 years with high and low familial likelihood of autism.

## Introduction

Executive functions (EFs), a diverse set of cognitive abilities that allow individuals to engage in goal-directed behavior, are critical to success in everyday life.^1,2,3^ Deficits in EF are associated with suboptimal outcomes, including poor academic, adaptive, and mental health functioning.^1,3,4^ Deficits in EF are more prevalent in neurodevelopmental disorders, including autism, a heritable condition characterized by social communication impairments and restricted and repetitive behaviors.^5^ Younger siblings of children with autism (denoted high familial likelihood [HL]) are at elevated likelihood of receiving an autism diagnosis compared with children who do not have a family history in first-degree relatives or an older sibling with autism (denoted low familial likelihood [LL]).^6^ Individuals with a HL of autism also exhibit a range of other neurodevelopmental and psychiatric outcomes that place them at high risk for EF difficulties.^7,8,9^ Early identification of subgroups at risk for EF deficits could expedite access to early intervention that improves executive dysfunction and associated disabling conditions.

Deficits in EF have been observed in children with autism as early as 24 months of age and are present across the life span.^10,11,12,13,14^ Familial studies of autism have also found EF deficits in HL siblings without autism on tasks of inhibition, cognitive flexibility, verbal and nonverbal fluency, and EF in everyday life,^15,16^ although these findings are not consistent across all studies.^17,18^ To our knowledge, the potential influence of biological sex on EF in autism has not been assessed early in development despite known sex differences in autism.

Autism is 4 times more prevalent in males than females.^19^ Genetics, sex hormones, and symptom presentation may contribute to sex differences in autism prevalence.^20,21,22^ Underrecognition of autism in females may occur because of bias in assessment criteria used to diagnose autism or ascertainment bias in studies that include females with autism.^23^ A previous study that used an infant sibling prospective design, in which participants were recruited before autism symptom onset, showed a more even sex ratio among boys and girls diagnosed with autism.^24^

In typical development, sex differences in EF have been observed at 24 months of age, with girls demonstrating better overall EF than boys. However, sex differences in EF have not been studied in children with autism younger than 6 years. In older samples, there is evidence that girls with autism have more impaired EF than boys with autism.^25^ Similarly, among HL siblings without autism, girls may have greater EF vulnerabilities than boys in specific domains (eg, cognitive flexibility).^26^

Brain structure differences could influence EF variability over and above differences in overall cerebral volume. A previous meta-analysis investigating neural activation in typically developing children found that regions within the frontal and parietal lobes were most consistently activated during EF tasks compared with other brain regions.^27^ Magnetic resonance imaging (MRI) studies in adolescents and adults with autism show activation across the frontoparietal regions but demonstrate an altered association with EF.^28,29,30,31^ No published studies, to our knowledge, have used structural MRI (sMRI) to elucidate brain-EF associations in very young children with autism or children at HL of autism despite evidence of cerebral enlargement in children with autism^32,33,34,35,36^ and frontoparietal abnormalities in HL siblings without autism.^37,38^

To address gaps in current knowledge about EF early in development in children with an older proband with autism, this study aimed to investigate the association of sex and autism likelihood group with EF in 24-month-old toddlers at HL and LL of autism and to examine whether specific brain alterations in the frontal and parietal regions, identified using sMRI, are associated with EF differences across sex and autism likelihood group. Differences in EF across sex and autism likelihood could elucidate potential familial aggregation of EF difficulties in autism.

## Methods

The Infant Brain Imaging Study is a longitudinal cohort study of brain and behavioral development in infants at HL for autism (with an older sibling with autism) and LL for autism (with an older sibling without autism). Participants were recruited at 4 clinical sites (Children’s Hospital of Philadelphia, University of Washington, University of North Carolina, and Washington University in St Louis) and were enrolled from January 1, 2007, to December 31, 2013. Older siblings of HL participants had an autism diagnosis confirmed by medical records and were above the cutoff for autism on the Social Communication Questionnaire^39^ and Autism Diagnostic Interview-Revised.^40^ Older siblings of LL participants fell below the cutoff score for autism on the Social Communication Questionnaire and had no first-degree relative with autism or intellectual disability. All participants were screened for exclusionary criteria: (1) birth weight less than 2000 g and/or gestational age less than 36 weeks or significant perinatal adversity and/or exposure in utero to neurotoxins; (2) medical or neurologic conditions affecting growth, development, or cognition or significant sensory impairments; (3) known genetic conditions or syndromes; (4) adopted or half-siblings or twins; (5) first-degree relative with significant psychiatric conditions (eg, schizophrenia); (6) contraindication for MRI; and (7) predominant home language other than English. All study procedures were approved by each site’s institutional review board (human participant division), and written informed consent was obtained from each participant’s parent. Executive function, developmental level, and autism symptoms were assessed at 24 months of age by expert clinicians to determine whether participants met criteria for autism spectrum disorder (ASD). See the eMethods in Supplement 1 for further details. Table 1 presents demographic and descriptive information. Participant race was identified by parent report during the screening interview to better characterize the sample. Groups did not differ on mean chronological age (in weeks), race, sex distribution, or maternal educational attainment (Table 1). The Strengthening the Reporting of Observational Studies in Epidemiology (STROBE) guidelines were followed in this study.

**Table 1.** Demographic Characteristics of the Study Sample^a^

Characteristic	HL for autism group		LL for autism group		*F* or χ^2^^b^	*P* value	Contrasts^c^
	Female (n = 45)	Male (n = 65)	Female (n = 30)	Male (n = 25)			
Age, mean (SD), mo	24.62 (0.90)	24.55 (0.92)	24.84 (1.09)	24.49 (0.95)	*F* = 0.80	.50	NA
ASD, No.	6	11	NA	NA	NA	NA	NA
Maternal educational attainment, No. (%)							
High school	15 (33)	25 (39)	5 (17)	5 (20)	χ^2^ = 7.49	.28	NA
College degree	20 (44)	26 (41)	14 (47)	12 (48)			
Graduate degree	10 (22)	13 (20)	11 (37)	8 (32)			
Race, No. (%)							
African American	0	0	1 (3)	0	χ^2^ = 1.45	.70	NA
Asian	0	2 (3)	1 (3)	1 (4)			
White	36 (80)	53 (82)	27 (90)	21 (84)			
>1 Race	9 (20)	10 (15)	1 (3)	3 (12)			
Mullen Early Learning Composite standard score, mean (SD)	103.13 (18.68)	98.55 (13.55)	115.17 (15.44)	104.6 (13.06)	*F* = 8.03	<.001	LL girls > HL girls
A-not-B total score, mean (% correct)	59.3007 (15.51)	55.5806 (17.87)	70.5506 (19.78)	65.4878 (19.55)	*F* = 5.46	.001	LL girls > HL girls
ADOS-2 Calibrated Severity Score, mean (SD)	2.0227 (1.78)	2.2769 (1.85)	1.3448 (0.61)	1.88 (1.24)	*F* = 2.34	.08	
Brain volumes, mean (SD), mm^3^^d^							
Total cerebrum	892 468 (86 867)	966 327 (73 747)	897 652 (75 581)	966 413 (80 627)	*F* = 8.24	<.001	HL boys > HL girls, LL boys > LL girls
Frontal lobe	309 233 (32 970)	330 544 (26 781)	309 582 (30 175)	332 848 (27 387)	*F* = 5.50	.001	HL boys > HL girls
Anterior frontal lobe	121 275 (14 082)	129 447 (11 879)	120 666 (10 159)	129 804 (11 893)	*F* = 4.70	.004	HL boys > HL girls
Posterior frontal lobe	187 958 (19 405)	201 096 (15 582)	188 916 (20 566)	203 044 (16 229)	*F* = 5.69	.001	HL boys > HL girls
Parietal lobe	239 360 (24 672)	260 675 (21 350)	238 789 (20 797)	258 138 (25 020)	*F* = 8.21	<.001	HL boys > HL girls

Abbreviations: ADOS-2, Autism Diagnostic Observation Schedule 2; ASD, autism spectrum disorder; HL, high familial likelihood; LL, low familial likelihood; NA, not applicable.

^a^ Some percentages do not equal 100% because of rounding.

^b^ The χ^2^ analysis was run on bifurcated race because of small numbers of participants of races other than White.

^c^ Only significant group differences (*P* < .05) are represented in this column. All other post hoc comparisons were nonsignificant.

^d^ Sample size for subset with brain volume data: n = 35 girls at HL for autism, n = 48 boys at HL for autism, n = 4 girls and 7 boys with ASD; n = 19 girls at LL for autism, and n = 18 boys at LL for autism.

### Executive Function

The A-not-B task assesses EF in infancy through preschool.^41,42,43^ In this task, toddlers watched as a toy was hidden to the left or right of midline in a well and were encouraged to find the toy after a 5-second delay. Once the hidden toy was found on 2 consecutive trials, the side of hiding was reversed and continued in this pattern until 3 sets of correct responses at a 5-second delay were successfully completed. The delay was then increased to 12 seconds. A maximum of 24 trials and 4 reversal trials were administered. Performance was measured by the proportion of total correct reaches by total trials.

### Covariates

Developmental level was selected as a covariate in this study and assessed with the Mullen Scales of Early Learning,^44^ a standardized, normed, developmental assessment for children from birth through 68 months of age. The Mullen Scales of Early Learning consists of 5 scales that assess early development with a composite standard score (Early Learning Composite standard score) used as a measure of overall developmental level. The standard score range was 49 to 155, with higher scores indicating better overall developmental functioning.

### Brain Imaging

Three-dimensional T1-weighted and 3-dimensional T2-weighted MRI images, with 1-mm^3^ voxel isotropic resolution, were acquired using a Siemens Trio 3T scanner at 24 months of age and used to determine lobular volumes based on a 24-month-old brain atlas.^35,45,46^ On the basis of the available literature, the frontal and parietal lobes were identified a priori as the primary anatomical regions of interest.^28,47^ The frontal lobe region comprised the frontal and prefrontal areas (coronal cut plane at the anterior of the corpus callosum) and adjacent cingulate white matter (WM) and gray matter (GM). The posterior frontal segment included the posterior portion of the frontal lobe (coronal cut plane at the anterior of the corpus callosum and the central sulcus) and adjacent cingulate. The parietal lobe region comprised parietal WM and GM and adjacent cingulate. The occipital lobe served as a comparison region and was composed of occipital WM and GM. Total cerebral volume included all WM and GM of the cerebrum and did not include the cerebellum, brainstem, or ventricles.

The MRI data were processed to obtain global and regional brain tissue volumes. Brain volume measurements were computed using an atlas-moderated expectation maximization segmentation, which includes rigid coregistration of multimodal (T1- and T2-weighted) MRI, bias correction, brain stripping, noise reduction, and multivariate classification with the AutoSeg toolkit, version 3.3.2 (NIRAL, University of North Carolina, Chapel Hill). Age-specific population average templates and corresponding probabilistic brain tissue priors for WM and GM were used. Regional and lobar parcellation of the brain was performed via single-template, multimodality deformable registration of a prior parcellation 24-month template (eFigure 1 in Supplement 1) via the Advanced Neuroimaging Tools toolkit (open source).^48^ The deformation field was then applied to the parcellation template and combined with the GM tissue segmentation, resulting in parcellated volumes in native space (eFigure 2 in Supplement 1).

### Statistical Analysis

A general linear model was used to evaluate associations between sex, autism likelihood group, and EF. This model consisted of EF as the outcome with sex, autism likelihood group, their interactions, and Mullen Early Learning Composite standard score as covariates. Estimated marginal means by sex and autism likelihood group from this model were estimated to examine group differences. General linear models were also used to further explore differences in brain-EF associations by autism likelihood group and sex. Separate models were used for frontal and parietal brain volumes. For each volume, these models consisted of EF as the outcome, sex, autism likelihood group, Early Learning Composite standard score, frontal or parietal volume, total cerebral volume, and all interactions among sex, autism likelihood group, and frontal or parietal volume as covariates. All brain volumes were centered in all analyses to a mean of zero. See the eMethods in Supplement 1 for further detail about statistical analysis. Because of the small sample in the HL group who met the diagnostic criteria for ASD (HL-ASD group; 6 girls and 11 boys), this group was not further subdivided by 24-month diagnostic outcome (ie, HL-ASD and HL-no-ASD were combined). All analysis were rerun without the HL-ASD group to assess whether ASD outcome was driving HL group differences, as well as controlling for maternal educational attainment. Given the limitations of multiple comparison correction in small samples,^49^ we instead used an uncorrected 2-sided *P* < .05 in combination with effect sizes to identify potentially meaningful differences. Combined with testing a small number of hypotheses, this strategy aimed to minimize the risk of type I error inflation with uncorrected *P* values. Effect sizes were interpreted according to the Cohen index (*R*^2^: ie, small = 0.02, medium = 0.15, and large = 0.35; η^2^_p_: small = 0.01, medium = 0.06, and large = 0.14).^50,51^ All analysis (performed between August 2021 and June 2022) were completed in SPSS software, version 27 (SPSS Inc) and in R software, version 3.6.0 (R Foundation for Statistical Computing).

## Results

### Association of Sex and Autism Likelihood Group With EF

A total of 165 toddlers (mean [SD] age, 24.61 [0.95] months; 90 boys [54%] and 75 girls [46%]; 1 African American [0.6%], 4 Asian [2.4%], 137 White [83%], and 23 of >1 race [13.9%]) were included in the study. The cohort included 110 toddlers at HL of autism (45 female and 65 male, 17 diagnosed with ASD) and 55 at LL of autism (30 female, 25 male).

Results are presented in Table 2. A significant difference was found in the A-not-B total score by autism likelihood group (B [SE] = −8.77 [4.21]; 95% CI, −17.09 to −0.45; η^2^_p_ = 0.03, *P* = .04) but not sex (η^2^_p_ = 0.00, *P* = .53), controlling for overall developmental level. The sex × autism likelihood group interaction was nonsignificant (η^2^_p_ = 0.00, *P* = .97). The planned sex × autism likelihood group contrasts revealed that boys and girls in the HL and LL groups scored similarly on the A-not-B task, but girls at HL of autism scored lower than girls at LL for autism, and boys at HL for autism scored lower than boys at LL for autism. With the exclusion of toddlers with autism, no group (HL vs LL) difference in A-not-B total score was found in males (mean [SE] difference = −7.18 [4.26]; 95% CI, 1.24 to 15.59) but the A-not-B total score was lower in HL girls than LL girls (mean [SE] difference = −9.75 [4.34]; 95% CI, −18.32 to −1.18) (eTable 1 in Supplement 1). Follow-up analyses evaluating the contribution of developmental level across sex and autism likelihood group to the model were nonsignificant (eTable 2 in Supplement 1).

**Table 2.** A-not-B Total Score by Sex and Likelihood for Autism Group Model Fit Results and Estimated Marginal Mean Differences^a^

Variable	Estimate (SE) [95% CI]	*P* value	*η^2^_p_*
Intercept	45.79 (10.13) [25.79 to 65.79]	<.001	0.11
Mullen Early Learning Composite standard score	0.19 (0.09) [0.01 to 0.37]	.04	0.03
Likelihood group (HL)	−8.77 (4.21) [−17.09 to −0.45]	.04	0.03
Sex (female)	3.07 (4.89) [−6.59 to 12.73]	.53	0.00
Likelihood group (HL) × sex (female)	−0.21 (5.93) [−11.92 to 11.50]	.97	0.00
Contrast			
HL girl vs LL girl	−8.98 (4.31) [−17.5 to −0.46]	.04	NA
HL girl vs HL boy	2.86 (3.47) [−3.99 to 9.71]	.41	NA
LL girl vs LL boy	3.07 (4.89) [−6.59 to 12.73]	.53	NA
HL boy vs LL boy	−8.77 (4.21) [−17.09 to −0.45]	.04	NA

Abbreviations: HL, high familial likelihood; LL, low familial likelihood; NA, not applicable.

^a^ *R*^2^/adjusted *R*^2^ = 0.12/0.09; F_4,159_ = 5.26; *P* = .001.

### Association of Sex, Autism Likelihood Group, and Cerebral Volume With EF

Results are presented in the Figure, Table 3, Table 4, and eTables 3 to 10 in Supplement 1. Comparing the results in Table 2 and Table 3, variance accounted for in the A-not-B total score increased by 6% when frontal lobe volume was added as a predictor (*R*^2^ = 0.12 vs 0.18). A significant sex × autism likelihood group × frontal lobe volume interaction was found after controlling for total cerebral volume and developmental level (η^2^_p_ = 0.04, *P* = .04) (Table 3). eTable 3 in Supplement 1 provides full model results when analyzed separately by sex and autism likelihood group. The frontal lobe × sex interaction was not statistically significant in the HL group (B [SE] = −1.36 [3.87]; 95% CI, −9.07 to 6.35; η^2^_p_ = 0.00, *P* = .73) but was significant in the LL group, with a medium effect size (B [SE] = 16.51 [7.43]; 95% CI, 1.36 to 31.67; η^2^_p_ = 0.14, *P* = .03). The frontal lobe × autism likelihood group interaction was significant in girls, with a medium effect size (B [SE] = −9.93 [4.89]; 95% CI, −19.73 to −0.12; η^2^_p_ = 0.08, *P* = .05), but not boys (B [SE] = 6.51 [5.88]; 95% CI, −5.26 to 18.27; η^2^_p_ = 0.02, *P* = .27). The results did not change when the model was rerun excluding the HL-ASD group (eTable 4 in Supplement 1). Anterior and posterior subdivisions of the frontal lobe were analyzed (eResults, eTables 5 and 6 in Supplement 1).

**Figure.**
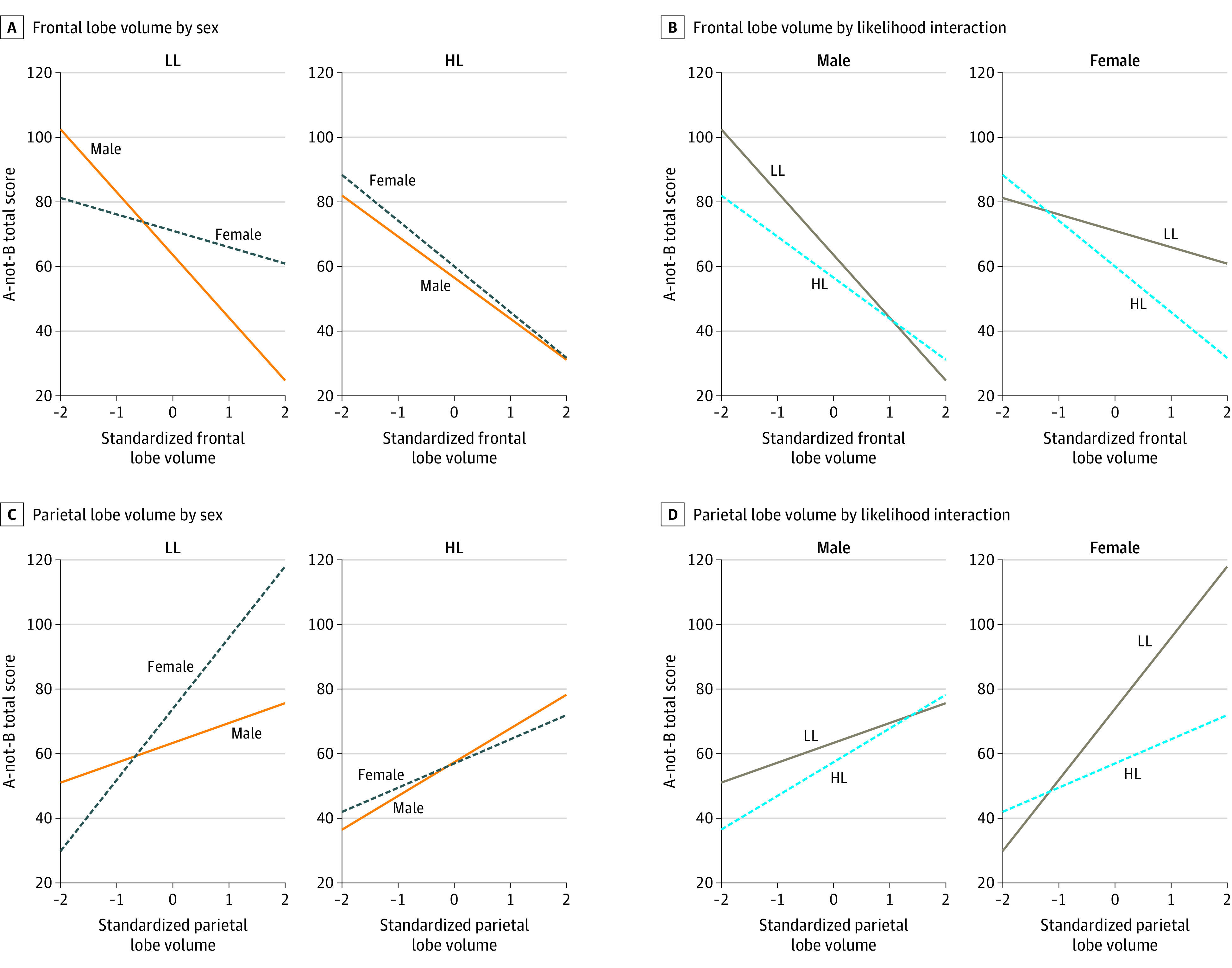
Frontal and Parietal Lobe Volume by Sex and Likelihood Group Interaction for A-not-B Total Score Higher A-not-B total score indicates better performance. A, The frontal lobe × sex interaction was not statistically significant in the high familial likelihood (HL) for autism group (*P* = .73) but was significant in the low familial likelihood (LL) for autism group (*P* = .03). B, The frontal lobe × likelihood interaction was significant in girls (*P* = .05) but not boys (*P* = .27). C, The parietal lobe × sex interaction was nonsignificant in the HL group (*P* = .49) but significant in the LL group (*P* = .02). D, The parietal lobe × likelihood group interaction was significant in girls (*P* = .005) but not boys (*P* = .45).

**Table 3.** A-not-B Total Score by Frontal Lobe Volume, Sex, and Likelihood of Autism Group Model Fit Results^a^

Variable	Estimate (SE) [95% CI]	*P* value	*η^2^_p_*
Intercept	37.54 (SE) [14.77 to 60.31]	.001	0.09
Mullen Early Learning Composite standard score	0.25 (0.11) [0.04 to 0.46]	.02	0.05
Total cerebrum	14.76 (6.18) [2.5 to 27.01]	.02	0.05
Total frontal lobe	−19.43 (7.86) [−35 to −3.86]	.01	0.05
Likelihood group (HL)	−7.04 (5.21) [−17.36 to 3.28]	.18	0.02
Sex (female)	7.47 (6.31) [−5.03 to 19.97]	.24	0.01
Total frontal lobe × likelihood group (HL)	6.72 (5.56) [−4.3 to 17.74]	.23	0.01
Total frontal lobe × sex (female)	14.35 (6.39) [1.68 to 27.02]	.03	0.04
Likelihood group (HL) × sex (female)	−3.99 (7.41) [−18.69 to 10.7]	.59	0.00
Total frontal lobe × likelihood group (HL) × sex (female)	−15.8 (7.57) [−30.81 to −0.78]	.04	0.04

Abbreviation: HL, high familial likelihood for autism.

^a^ *R*^2^/adjusted *R*^2^ = 0.18/0.11; F_9,109_ = 2.58; *P* = .01.

**Table 4.** A-not-B Total Score by Parietal Lobe Volume, Sex, and Likelihood of Autism Group Model Fit Results^a^

Variable	Estimate (SE) [95% CI]	*P* value	*η^2^_p_*
Intercept	36.66 (11.36) [14.15 to 59.18]	.002	0.09
Mullen Early Learning Composite standard score	0.26 (0.10) [0.05 to 0.46]	.02	0.05
Total cerebrum	−8.28 (5.36) [−18.91 to 2.35]	.13	0.02
Total parietal lobe	6.14 (6.34) [−6.43 to 18.71]	.33	0.01
Likelihood group (HL)	−5.98 (5.10) [−16.08 to 4.12]	.24	0.01
Sex (female)	10.53 (6.32) [−2.00 to 23.07]	.10	0.03
Total parietal lobe × likelihood group (HL)	4.29 (5.05) [−5.71 to 14.29]	.40	0.01
Total parietal lobe × sex (female)	15.85 (6.38) [3.20 to 28.51]	.01	0.05
Likelihood group (HL) × sex (female)	−10.93 (7.52) [−25.84 to 3.98]	.15	0.02
Total parietal lobe × likelihood group (HL) × sex (female)	−18.8 (7.65) [−33.97 to −3.64]	.02	0.05

Abbreviation: HL, high familial likelihood for autism.

^a^ *R*^2^/adjusted *R*^2^ = 0.19/0.12; F_9,108_ = 2.77; *P* = .006.

Comparing the results in Table 2 and Table 4, variance in the A-not-B total score increased by 7% when parietal lobe volume was added as a predictor to the model (*R*^2^ = 0.12 vs 0.19). There was a significant sex × autism likelihood group × parietal lobe interaction. The parietal lobe × sex interaction was nonsignificant in the HL group (B [SE] = −2.81 [4.09]; 95% CI, −10.96 to 5.34; η^2^_p_ = 0.01, *P* = .49) but statistically significant in the LL group, with a large effect size (B [SE] = 17.68 [6.99]; 95% CI, 3.42-31.94; η^2^_p_ = 0.17, *P* = .02). There was also a significant parietal lobe × autism likelihood group interaction in girls, with a large effect size (B [SE] = −15.44 [5.18]; 95% CI, −25.86 to −5.02; η^2^_p_ = 0.16, *P* = .005), but not in boys (B [SE] = 4.18 [5.48]; 95% CI, −6.78 to 15.15; η^2^_p_ = 0.01, *P* = .45). eTable 7 in Supplement 1 provides full model results. Removal of the HL-ASD group did not change the results (eTable 4 in Supplement 1).

Given the association between maternal educational attainment and brain growth,^52^ frontal and parietal lobe models were rerun to include maternal educational attainment as a covariate, which did not change the overall results (eTables 8 and 9 in Supplement 1). The overall model was nonsignificant when including the occipital lobe control region (eTable 10 in Supplement 1).

## Discussion

Differences in EF were associated with autism likelihood group, sex, and frontal and parietal lobe brain volume at 24 months of age. Consistent with a prior report,^12^ we found that toddlers at HL of autism demonstrated divergent EF development by 24 months of age compared with toddlers at LL of autism. Additionally, this is the first report of which we are aware to suggest that EF may be related to biological sex and brain lobular volume in autism. When girls and boys were compared within a given autism likelihood group, no male-vs-female EF differences were found. However, girls and boys in the HL group demonstrated worse EF than their same-sex LL counterparts. In girls, autism likelihood group differences in EF remained significant when girls in the HL-ASD group were excluded; however, in boys, EF differences appeared to be driven by the HL-ASD subgroup. Follow-up analysis suggested that these EF differences were not attributable to developmental level.

Prior studies using a familial design to study EF in other conditions, such as attention-deficit/hyperactivity disorder and obsessive compulsive disorder, have suggested that EF deficits may be heritable.^53,54^ Similarly, in autism, which is a highly heritable condition, EF deficits have been observed in relatives of children with autism.^15,16^ Consistent with these studies,^53,54^ our data support the possibility that EF deficits may aggregate in families in which there is an older proband with autism. Furthermore, our data demonstrated differences between girls without autism in the HL and LL groups but not boys without autism in the HL and LL groups, perhaps suggesting that sex differences in familial aggregation of EF deficits could exist. However, heritability of EF deficits was not directly tested. Additionally, it is possible that power to detect differences between boys in the HL and LL groups was decreased after removal of the HL-ASD group because more boys had autism. Therefore, further exploration of the influence of sex on the heritability of EF deficits is needed.

In contrast to prior research demonstrating a female EF advantage in typically developing samples^55,56,57^ and male EF advantage in siblings at HL of autism,^26^ we found no male or female EF advantage in either the HL or LL groups. An important contributor to the differences in our findings compared with prior literature may be the age at which sex differences in EF emerge.^58,59^ Our 24-month-old cohort is the youngest sample of HL siblings with and without autism studied to date. Viewed from a developmental context, lack of sex differences in EF could indicate that male-vs-female sex differences, as reported in the literature on older individuals, emerge between preschool and school-age.^26,55^ In addition, because of the young age of our sample, we used a developmentally appropriate EF task not used with older children or adults. Thus, task differences, a known source of variability across studies, could also contribute to differences in our findings.^60^ Prior research indicates that sex differences may be domain specific, with studies finding sex differences in some domains (eg, visuospatial working memory) but not others (eg, decision making),^58,59^ which may also be relevant to our findings. For example, Bölte et al^26^ found a male advantage in cognitive flexibility in siblings at HL of autism, whereas we found no sex-based EF advantage on our task tapping EF more broadly.

Our findings also indicate that larger lobular volume is associated with decreased EF performance in the frontal lobe and increased EF performance in the parietal lobe, even when accounting for total cerebral volume and developmental level. Furthermore, our findings suggest that boys (regardless of likelihood group) and girls at HL of autism showed less resilience in EF as frontal lobe volume increased and less improvement in EF as parietal lobe volume increased than girls at LL of autism. Given the general paucity of research on brain-behavior EF relationships in toddlers at HL of autism, it is difficult to compare our findings to previous work. It is possible that differences in cortical growth and maturation through the process of GM arborization and WM myelination could drive differences in EF brain-behavior associations.^61,62,63^ Furthermore, there is evidence that brain maturation progresses in a posterior to anterior pattern, with the parietal lobe maturing sooner than the frontal lobe.^61,64^ This relative difference in maturation may, in part, account for variable EF-brain associations in the frontal and parietal lobes across groups. In addition, maturation in these regions generally occurs earlier in girls than boys^63,65^ and might explain why EF–frontal lobe and EF–parietal lobe associations were more divergent among girls than boys in our sample. Future longitudinal studies will be needed to map the sex-specific EF brain-behavior developmental trajectories in male and female children at HL of autism.

### Limitations

Several limitations in the current study should be acknowledged. Because of small sample sizes in the HL-ASD subgroup, we were unable to assess sex × diagnostic group differences in EF. The sample sizes for girls and individuals of races other than White were also relatively small, and future studies with larger samples of HL-ASD siblings, girls, and racially underrepresented children are needed to evaluate whether the current findings are generalizable. However, our sample was unbiased with respect to ascertainment of girls with autism.^24^ Results presented in this article should be considered preliminary and interpreted with caution because of possible inflation of type I error because no correction for multiple comparisons was made. However, we believe that this less conservative approach has generated novel hypotheses that warrant further investigation. Future studies should expand brain and behavior measurement batteries. Multiple measures of EF could add specificity to the behavior and brain-based associations observed in the current study.^66^ Additional imaging techniques (eg, diffusion tensor imaging and functional connectivity MRI) could further elucidate underlying mechanisms associated with EF, such as WM structure and functional connections between brain regions, which will be explored in future work.

## Conclusions

This cohort study of 24-month-old children found that EF differences were associated with sex, autism likelihood, and frontal and parietal lobe volume. These findings suggest that sex differences in familial aggregation of EF deficits could exist and highlight the importance of investigating the influence of sex in future brain-behavior studies of EF. Additional research connecting early, sex-based differences in EF development to later outcomes is also warranted.

## Notes

Supplement 1. **eMethods.** Supplementary Methods **eResults.** Supplementary Results **eFigure 1.** 24-Month-Old Segmentation Template **eFigure 2.** 24-Month-Old Case Example of Segmentation Results **eTable 1.** Estimated Marginal Mean Differences in A-not-B Total Score by Sex and Likelihood Group *Without* HL-ASD Group **eTable 2.** A-not-B Total Score by Sex, Likelihood Group, and Developmental Level Model Fit Results **eTable 3.** Model Fit Results for A-not-B Total Score by Frontal Lobe Separated by Sex and Likelihood Group **eTable 4.** Brain Volume by Sex by Likelihood Group Interaction for A-not-B Total Score With HL-ASD Group Removed **eTable 5.** A-not-B Total Score by Anterior Frontal Lobe Volume, Sex, and Likelihood Group Model Fit Results **eTable 6.** A-not-B by Posterior Frontal Lobe Volume, Sex, and Likelihood Group Model Fit Results **eTable 7.** Model Fit Results for A-not-B Total Score by Parietal Lobe Separated by Sex and Likelihood Group **eTable 8.** A-not-B Total Score by Frontal Lobe, Sex and Likelihood Group Model Fit Results (With Maternal Education) **eTable 9.** A-not-B Total Score by Parietal Lobe, Sex and Likelihood Group Model Fit Results (With Maternal Education) **eTable 10.** A-not-B Total Score by Occipital Lobe, Sex and Likelihood Group Model Fit Results (With Maternal Education)
eReferences

Supplement 2.
Nonauthor Collaborators

Supplement 3.
Data Sharing Statement
