# Supplementary material for: Association of Sex With Neurobehavioral Markers of Executive Function in 2-Year-Olds at High and Low Likelihood of Autism
Source: JAMA Netw Open. 2023 May 4;6(5):e2311543. doi: 10.1001/jamanetworkopen.2023.11543 (PMC10160873; doi:10.1001/jamanetworkopen.2023.11543)
Supplement: Supplement 1. — eMethods. Supplementary Methods eResults. Supplementary Results eFigure 1. 24-Month-Old Segmentation Template eFigure 2. 24-Month-Old Case Example of Segmentation Results eTable 1. Estimated Marginal Mean Differences in A-not-B Total Score by Sex and Likelihood Group Without HL-ASD Group eTable 2. A-not-B Total Score by Sex, Likelihood Group, and Developmental Level Model Fit Results eTable 3. Model Fit Results for A-not-B Total Score by Frontal Lobe Separated by Sex and Likelihood Group eTable 4. Brain Volume by Sex by Likelihood Group Interaction for A-not-B Total Score With HL-ASD Group Removed eTable 5. A-not-B Total Score by Anterior Frontal Lobe Volume, Sex, and Likelihood Group Model Fit Results eTable 6. A-not-B by Posterior Frontal Lobe Volume, Sex, and Likelihood Group Model Fit Results eTable 7. Model Fit Results for A-not-B Total Score by Parietal Lobe Separated by Sex and Likelihood Group eTable 8. A-not-B Total Score by Frontal Lobe, Sex and Likelihood Group Model Fit Results (With Maternal Education) eTable 9. A-not-B Total Score by Parietal Lobe, Sex and Likelihood Group Model Fit Results (With Maternal Education) eTable 10. A-not-B Total Score by Occipital Lobe, Sex and Likelihood Group Model Fit Results (With Maternal Education) eReferences [file jamanetwopen-e2311543-s001.pdf]

# Supplementary Online Content

St. John T, Estes AM, Hazlett HC, et al; IBIS Network. Association of sex with neurobehavioral markers of executive function in 2-year-olds at high and low likelihood of autism. *JAMA Netw Open*. 2023;6(5):e2311543. doi:10.1001/jamanetworkopen.2023.11543

**eMethods.** Supplementary Methods
**eResults.** Supplementary Results
**eFigure 1.** 24-Month-Old Segmentation Template
**eFigure 2.** 24-Month-Old Case Example of Segmentation Results
**eTable 1.** Estimated Marginal Mean Differences in A-not-B Total Score by Sex and Likelihood Group *Without* HL-ASD Group
**eTable 2.** A-not-B Total Score by Sex, Likelihood Group, and Developmental Level Model Fit Results
**eTable 3.** Model Fit Results for A-not-B Total Score by Frontal Lobe Separated by Sex and Likelihood Group
**eTable 4.** Brain Volume by Sex by Likelihood Group Interaction for A-not-B Total Score With HL-ASD Group Removed
**eTable 5.** A-not-B Total Score by Anterior Frontal Lobe Volume, Sex, and Likelihood Group Model Fit Results
**eTable 6.** A-not-B by Posterior Frontal Lobe Volume, Sex, and Likelihood Group Model Fit Results
**eTable 7.** Model Fit Results for A-not-B Total Score by Parietal Lobe Separated by Sex and Likelihood Group
**eTable 8.** A-not-B Total Score by Frontal Lobe, Sex and Likelihood Group Model Fit Results (With Maternal Education)
**eTable 9.** A-not-B Total Score by Parietal Lobe, Sex and Likelihood Group Model Fit Results (With Maternal Education)
**eTable 10.** A-not-B Total Score by Occipital Lobe, Sex and Likelihood Group Model Fit Results (With Maternal Education)
**eReferences**

This supplementary material has been provided by the authors to give readers additional information about their work.

© 2023 St. John T et al. *JAMA Network Open.*

**eMethods.** Supplementary Methods

**Procedures**

Participants were recruited at four clinical sites (Children's Hospital of Philadelphia, University of Washington, University of North Carolina, and Washington University) through flyers, brochures, community clinics and organizations, and research registries. All study procedures were approved by each site's Human Subjects Division, Institutional Review Board and written informed consent was obtained from each participant's parent. Participants were evaluated at 6 months, 12 months, and 24 months of age. Data for the current study is focused on the 24-month timepoint (range = 22 months - 31months of age). At 24 months, EF and overall developmental level were assessed by a licensed clinical psychologist, doctoral student in clinical psychology, school psychologist, or masters-level psychometrist under supervision of a licensed clinical psychologist or child psychiatrist. Clinical best estimates for ASD using DSM-IV-TR criteria were made using all available clinical information (e.g., Autism Diagnostic Observation Schedule[1], Autism Diagnostic Interview-Revised[2]).

Two MRI acquisitions failed rigorous quality control, both due to severe ringing artefacts. However, there were additional reasons for missing MRI data that include failed studies due to the infant waking up during scanning (i.e., incomplete acquisitions), the infant being unable to fall asleep when the MRI was attempted, or parents declining the MRI. Participants without MRI data did not differ significantly in age, maternal education, overall developmental level, race, or likelihood group from participants with MRI data (all $P > .05$).

**Statistical Analysis**

Model residuals were visually inspected for normality and outliers using per-model QQ-plots and histograms. Per-observation leverage statistics were also computed and checked to verify no observation had an outsized impact on the model fit. No outliers in the residuals were identified.

© 2023 St. John T et al. *JAMA Network Open.*

**eResults** Supplementary Results

Comparing the results in Table 2 and eTable 5 and eTable 6, variance explained in the A-not-B total score increased by 7% when anterior frontal lobe volume was added as a predictor ($R^2$ = 0.12 vs. $R^2$ = 0.18) and marginally increased (3%) when posterior frontal lobe volume was added as a predictor ($R^2$ = 0.12 vs. $R^2$ = 0.15). There was a significant sex by autism likelihood group by anterior frontal lobe interaction (eTable 5). The anterior frontal lobe by sex interaction was not statistically significant in the HL group ($\eta^2_p$ = 0.00, *p*=.64) but significant in the LL group with a medium to large effect ($\eta^2_p$ = 0.12., *p*=.04). The anterior frontal lobe by autism likelihood group interaction in females was significant ($\eta^2_p$ = 0.08, *p*=.05) and in males was non-significant ($\eta^2_p$ = 0.03, *p*=.16). The anterior frontal lobe by autism likelihood group interaction in females remained significant after the HL-ASD group was removed ($\eta^2_p$ = 0.11, *p*=.024; eTable 4), the results did not otherwise change. No significant interactions for the posterior lobe model were found (eTable 6). After the HL-ASD group was removed, the sex by autism likelihood group by posterior-frontal lobe volume interaction was significant ($\eta^2_p$ =.04, *p*=.04, eTable 4) but there were no posterior-frontal lobe volume by sex interactions in the HL group ($\eta^2_p$ = 0.00, *p*=.74) or LL group ($\eta^2_p$ = 0.09, *p*=.08) nor posterior-frontal lobe by autism likelihood group interaction in males ($\eta^2_p$ = 0.01, *p*=.44). However, the posterior-frontal lobe by autism likelihood interaction in females, excluding the HL-ASD group, was significant with a medium effect ($\eta^2_p$ = 0.13, *p*=.01).

© 2023 St. John T et al. *JAMA Network Open.*

**eFigure 1. 24-month Segmentation Template**

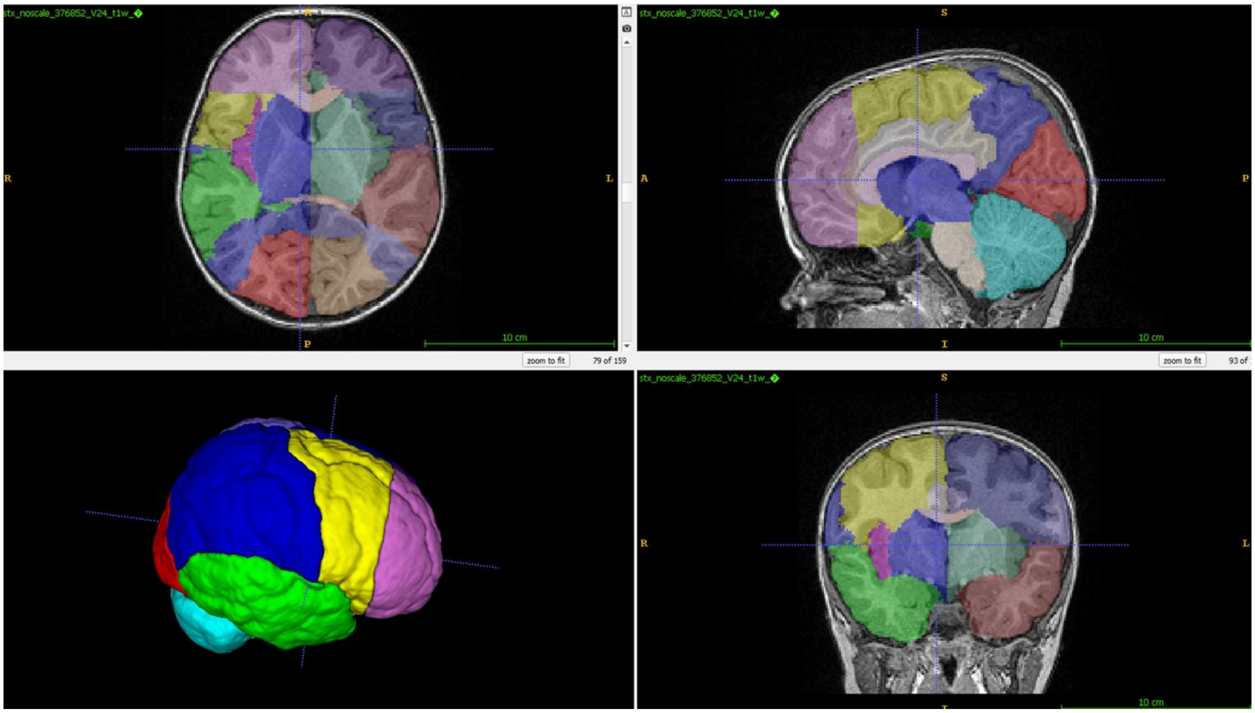

eFigure 1. Regional/lobar parcellation of the brain was performed with single-template, multi-modality deformable registration of a prior parcellation 24-month template using the Advanced Neuroimaging Tools (ANTs) toolkit.[3] Specific details of our segmentation approach are described in Kim et al.[4] Actual example shown in eFigure 2.

**eFigure 2. 24-month-old Case Example of Segmentation Result**

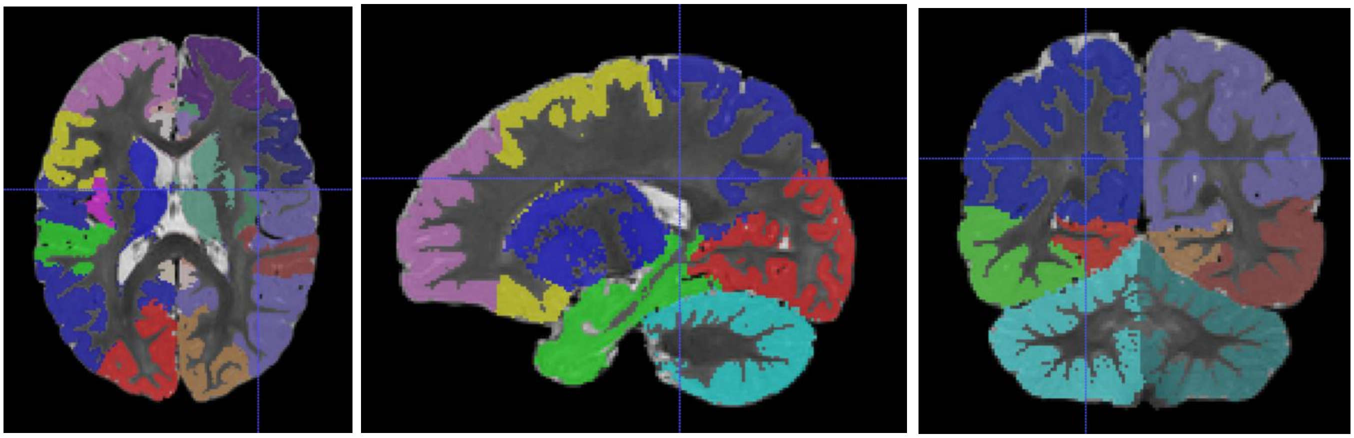

© 2023 St. John T et al. *JAMA Network Open.*

eTable 1. Estimated Marginal Mean Differences in A-not-B Total Score by Sex and Likelihood Group *without* HL-ASD Group

| Contrast | Estimate (95% CI) | SE | *P*-value |
|---|---|---|---|
| HL Female-HL Male | 0.30 (-7.06, 7.66) | 3.72 | .94 |
| LL Female-LL Male | 2.87 (-6.70, 12.44) | 4.84 | .55 |
| HL Female-LL Female | -9.75 (-18.32, -1.18) | 4.34 | .03 |
| HL Male-LL Male | -7.18 (-1.24, 15.59) | 4.26 | .09 |

Abbreviations: HL, high familial likelihood for autism; LL, low familial likelihood for autism

© 2023 St. John T et al. *JAMA Network Open.*

eTable 2. A-not-B Total Score by Sex, Likelihood Group, and Developmental Level Model Fit Results

| | Estimate (95% CI) | SE | *P*-value | $\eta^2_p$ |
|---|---|---|---|---|
| Intercept | 19.29 (-38.58 to 77.15) | 29.29 | .51 | 0.00 |
| Mullen Early Learning Composite | 0.44 (-0.11 to 0.99) | 0.28 | .11 | 0.02 |
| Sex (Female) | 22.14 (-44.09 to 88.38) | 33.53 | .51 | 0.00 |
| Likelihood Group (HL) | 49.68 (-26.19 to 125.55) | 38.41 | .20 | 0.01 |
| Sex (Female):Likelihood Group (HL) | -55.92 (-143.54 to 31.71) | 44.36 | .21 | 0.01 |
| Sex (Female):Mullen Early Learning Composite | -0.43 (-1.12 to 0.27) | 0.35 | .22 | 0.01 |
| Likelihood Group (HL):Mullen Early Learning Composite | -0.30 (-0.94 to 0.34) | 0.32 | .36 | 0.01 |
| Sex (Female):Likelihood Group (HL):Mullen Early Learning Composite | 0.52 (-0.30 to 1.33) | 0.41 | .21 | 0.01 |

Abbreviations: HL, high familial likelihood for autism; LL, low familial likelihood for autism
$R^2$/Adjusted $R^2$ = 0.13/0.09
F(7,156)= 3.22, *P* = .003

© 2023 St. John T et al. *JAMA Network Open.*

eTable 3. Model Fit Results for A-not-B Total Score by Frontal Lobe Separated by Sex and Likelihood Group

| | Estimate (95% CI) | SE | *P*-value | $\eta^2_p$ |
|---|---|---|---|---|
| **Female[a]** | | | | |
| Intercept | 35.98 (4.54, 67.42) | 15.64 | .03 | 0.10 |
| Mullen Early Learning Composite | 0.33 (0.07, 0.60) | 0.13 | .02 | 0.12 |
| Likelihood Group (HL) | -10.40 (-20.55, -0.25) | 5.05 | .05 | 0.08 |
| Total Cerebrum | 17.76 (-1.89, 37.40) | 9.77 | .08 | 0.06 |
| Total Frontal Lobe | -7.56 (-26.298, 11.18) | 9.32 | .42 | 0.01 |
| Likelihood Group (HL) * Total Frontal Lobe | -9.93 (-19.73, -0.12) | 4.88 | .05 | 0.08 |
| **Male[b]** | | | | |
| Intercept | 47.15 (11.92, 82.37) | 17.60 | .01 | 0.11 |
| Mullen Early Learning Composite | 0.157 (-0.181, 0.494) | 0.17 | .36 | 0.01 |
| Likelihood Group (HL) | -7.33 (-18.43, 3.780 | 5.55 | .19 | 0.03 |
| Total Cerebrum | 14.02 ( -2.40, 30.45) | 8.21 | .09 | 0.05 |
| Total Frontal Lobe | -18.15 (-37.45, 1.14) | 9.64 | .07 | 0.06 |
| Likelihood Group (HL) * Total Frontal Lobe | 6.51 ( -5.26, 18.27) | 5.88 | .27 | 0.02 |
| **HL[c]** | | | | |
| Intercept | 34.72 (11.99, 57.45) | 11.41 | .003 | 0.11 |
| Mullen Early Learning Composite | 0.21 (-0.02, 0.44) | 0.12 | .07 | 0.04 |
| Sex (Female) | 3.33 (-5.44, 12.11) | 4.40 | .45 | 0.01 |
| Total Cerebrum | 12.27 (-1.88, 26.41) | 7.10 | .09 | 0.04 |
| Total Frontal Lobe | -10.22 (-24.57, 4.12) | 7.21 | .16 | 0.03 |
| Sex (Female):Total Frontal Lobe | -1.36 (-9.07, 6.35) | 3.87 | .73 | 0.00 |
| **LL[d]** | | | | |
| Intercept | 21.06 (-31.00, 73.12) | 25.53 | .42 | 0.02 |
| Mullen Early Learning Composite | 0.42 (-0.09, 0.92) | 0.25 | .11 | 0.08 |
| Sex (Female) | 5.98 (-9.40, 21.36) | 7.54 | .43 | 0.02 |
| Total Cerebrum | 21.64 (-3.92, 47.20) | 12.53 | .09 | 0.09 |
| Total Frontal Lobe | -27.32 (-55.70, 1.06) | 13.91 | .06 | 0.11 |
| Sex (Female):Total Frontal Lobe | 16.51 (1.36, 31.67) | 7.43 | .03 | 0.14 |

Abbreviations: HL, high familial likelihood for autism; LL, low familial likelihood for autism

[a] $R^2$ /$R^2$ Adjusted=.27/.19

[b] $R^2$/$R^2$ Adjusted=.09/.01

[c] $R^2$/$R^2$ Adjusted=.09/.03

[d] $R^2$/$R^2$ Adjusted= .24/.12

© 2023 St. John T et al. *JAMA Network Open.*

eTable 4. Brain Volume by Sex by Likelihood Group Interaction for A-not-B Total Score with HL-ASD group removed

| | Estimate (95% CI) | SE | *P*-value | $\eta^2_p$ |
|---|---|---|---|---|
| Sex (Female):Likelihood Group (HL):Total Frontal Lobe | -18.82 (-33.21 to -3.15) | 7.57 | .02 | 0.06 |
| Sex (Female):Likelihood Group (HL):Total Anterior Frontal Lobe | -20.10 (-35.64 to -4.55) | 7.84 | .01 | 0.06 |
| Sex (Female):Likelihood Group (HL):Total Posterior Frontal Lobe | -16.12 (-31.29 to -.97) | 7.64 | .04 | 0.04 |
| Sex (Female):Likelihood Group (HL);Total Parietal Lobe | -21.03 (-36.17 to -5.89) | 7.63 | .007 | 0.07 |

Abbreviations: HL, high familial likelihood for autism; LL, low familial likelihood for autism

© 2023 St. John T et al. *JAMA Network Open.*

eTable 5. A-not-B Total Score by Anterior Frontal Lobe Volume, Sex, and Likelihood Group Model Fit Results

| | Estimate (95% CI) | SE | *P*-value | $\eta^2_p$ |
|---|---|---|---|---|
| Intercept | 35.72 (12.83, 58.61) | 11.55 | .003 | 0.08 |
| Mullen Early Learning Composite | 0.26 (0.05, 0.47) | 0.11 | .02 | 0.05 |
| Sex (Female) | 8.45 (-4.03, 20.93) | 6.30 | .18 | 0.02 |
| Likelihood Group (HL) | -6.26 (-16.26, 3.74) | 5.05 | .22 | 0.01 |
| Total Cerebrum | 12.21 (1.10, 23.33) | 5.61 | .03 | 0.04 |
| Total Anterior Frontal Lobe | -17.22 (-31.23, -3.20) | 7.07 | .02 | 0.05 |
| Sex (Female):Likelihood Group (HL) | -4.64 (-19.42, 10.14) | 7.46 | .54 | 0.00 |
| Sex (Female):Total Anterior Frontal Lobe | 15.75 (2.16, 29.33) | 6.85 | .02 | 0.05 |
| Likelihood Group (HL):Total Anterior Frontal Lobe | 7.49 (-2.96, 17.94) | 5.27 | .16 | 0.02 |
| Sex (Female):Likelihood Group (HL):Total Anterior Frontal Lobe | -17.60 (-33.2, -1.99) | 7.88 | .03 | 0.04 |

Abbreviations: HL, high familial likelihood for autism; LL, low familial likelihood for autism
[a] $R^2$ /$R^2$ Adjusted = 0.18/0.12,
$F(9, 109) = 2.72$, $P = .007$

© 2023 St. John T et al. *JAMA Network Open.*

eTable 6. A-not-B Total Score by Posterior Frontal Lobe Volume, Sex, and Likelihood Group Model Fit Results

| | Estimate (95% CI) | SE | *P*-value | $\eta^2_p$ |
|---|---|---|---|---|
| Intercept | 39.12 (16.06, 62.17) | 11.63 | .001 | 0.09 |
| Mullen Early Learning Composite | 0.24 (0.02, 0.45) | 0.11 | .030 | 0.04 |
| Sex (Female) | 7.00 (-5.72, 19.73) | 6.42 | .278 | 0.01 |
| Likelihood Group (HL) | -6.38 (-16.97, 4.21) | 5.34 | .235 | 0.01 |
| Total Cerebrum | 8.85 (-0.85, 18.54) | 4.89 | .073 | 0.03 |
| Total Posterior Frontal Lobe | -12.19 (-25.82, 1.44) | 6.88 | .079 | 0.03 |
| Sex (Female):Likelihood Group (HL) | -4.82 (-19.69, 10.05) | 7.50 | .522 | 0.00 |
| Sex (Female):Total Posterior Frontal Lobe | 11.49 (-1.01, 23.99) | 6.31 | .071 | 0.03 |
| Likelihood Group (HL):Total Posterior Frontal Lobe | 5.07 (-6.39, 16.53) | 5.78 | .382 | 0.01 |
| Sex (Female):Likelihood Group (HL):Total Posterior Frontal Lobe | -12.93 (-28.03, 2.18) | 7.62 | .093 | 0.03 |

Abbreviations: HL, high familial likelihood for autism; LL, low familial likelihood for autism
[a] $R^2$ /$R^2$ Adjusted = 0.15/0.08
$F(9, 109) = 2.12$, $P = .03$

© 2023 St. John T et al. *JAMA Network Open.*

eTable 7. Model Fit Results for A-not-B Total Score by Parietal Lobe Separated by Sex and Likelihood Group

| | Estimate (95% CI) | SE | *P*-value | $\eta^2_p$ |
|---|---|---|---|---|
| **Female**[a] | | | | |
| Intercept | 38.09 (8.93, 67.23) | 14.49 | .012 | 0.13 |
| Mullen Early Learning Composite | 0.34 (0.09, 0.59) | 0.12 | .009 | 0.14 |
| Likelihood Group (HL) | -17.12 (-27.45, -6.83) | 5.12 | .002 | 0.19 |
| Total Cerebrum | -12.72 (-26.33, 0.86) | 6.76 | .07 | 0.07 |
| Total Parietal Lobe | 26.55 (10.84, 42.24) | 7.80 | .001 | 0.20 |
| Likelihood Group (HL) * Total Parietal Lobe | -15.44 (-25.86, -5.02) | 5.18 | .005 | 0.16 |
| **Male**[b] | | | | |
| Intercept | 46.51 (10.53, 82.50) | 17.98 | .01 | 0.10 |
| Mullen Early Learning Composite | 0.16 (-0.19, 0.5) | 0.17 | .37 | 0.01 |
| Likelihood Group (HL) | -6.03 (-17.24, 5.18) | 5.60 | .29 | 0.02 |
| Total Cerebrum | -3.96 (-20.41, 12.49) | 8.22 | .63 | 0.00 |
| Total Parietal Lobe | 2.65 (-14.68, 19.970 | 8.66 | .76 | 0.00 |
| Likelihood Group (HL) * Total Parietal Lobe | 4.18 (-6.78, 15.15) | 5.48 | .45 | 0.01 |
| **HL**[c] | | | | |
| Intercept | 35.98 (13.20, 58.76) | 11.44 | .002 | 0.12 |
| Mullen Early Learning Composite | 0.20 (-0.03, 0.43) | 0.12 | .08 | 0.04 |
| Sex (Female) | 0.56 (-8.25, 9.36) | 4.42 | .90 | 0.00 |
| Total Cerebrum | -5.11 (-17.63, 7.42) | 6.29 | .42 | 0.01 |
| Total Parietal Lobe | 7.70(-5.29, 20.65) | 6.50 | .24 | 0.02 |
| Sex (Female) * Total Parietal Lobe | -2.81 (-10.96, 5.34) | 4.09 | .49 | 0.00 |
| **LL**[d] | | | | |
| Intercept | 17.90 (-32.06, 67.86) | 24.49 | .47 | 0.02 |
| Mullen Early Learning Composite | 0.45 (-0.04, 0.94) | 0.24 | .07 | 0.10 |
| Sex (Female) | 7.87 (-6.73, 22.47) | 7.16 | .28 | 0.04 |
| Total Cerebrum | -15.67 (-36.66, 5.32) | 10.29 | .12 | 0.07 |
| Total Parietal Lobe | 12.037 (-8.85, 32.93) | 10.24 | .25 | 0.04 |
| Sex (Female) * Total Parietal Lobe | 17.68 (3.43, 31.94) | 6.99 | .02 | 0.17 |

Abbreviations: HL, high familial likelihood for autism; LL, low familial likelihood for autism
[a] $R^2$ /$R^2$ Adjusted= .36/.29
[b] $R^2$ /$R^2$ Adjusted= .05/-.03
[c] $R^2$ /$R^2$ Adjusted=.07/.01
[d] $R^2$ /$R^2$ Adjusted=.31/.19

© 2023 St. John T et al. *JAMA Network Open.*

eTable 8. A-not-B Total Score by Frontal Lobe, Sex and Likelihood Group Model Fit Results (with Maternal Education)

| | Estimate (95% CI) | SE | *P*-value | $\eta^2_p$ |
|---|---|---|---|---|
| Intercept | 26.42 (-2.25, 55.09) | 14.44 | .07 | 0.03 |
| Mullen Early Learning Composite | 0.34 (0.10 , 0.58) | 0.12 | .005 | 0.08 |
| Maternal Education (High School)[a] | 3.41 (-7.00, 13.83) | 5.25 | .52 | 0.00 |
| Maternal Education (College)[a] | 1.42 (-7.86, 10.69) | 4.67 | .76 | 0.00 |
| Sex (Female) | 6.53 (-5.889, 18.94) | 6.25 | .30 | 0.01 |
| Likelihood Group (HL) | -5.35 (-15.83, 5.12) | 5.28 | .31 | 0.01 |
| Total Cerebrum | 11.36 (-1.50, 24.21) | 6.48 | .08 | 0.03 |
| Total Frontal Lobe | -15.91 (-32.15, 0.33) | 8.18 | .06 | 0.04 |
| Sex (Female):Likelihood Group (HL) | -7.23 (-22.14, 7.69) | 7.51 | .34 | 0.01 |
| Sex (Female):Total Frontal Lobe | 13.36 (0.52, 26.20) | 6.47 | .04 | 0.04 |
| Likelihood Group (HL):Total Frontal Lobe | 7.17 (-4.17, 18.51) | 5.71 | .21 | 0.02 |
| Sex (Female):Likelihood Group (HL):Total Frontal Lobe | -17.21 (-32.64, -1.78) | 7.77 | .03 | 0.05 |

Abbreviations: HL, high familial likelihood for autism; LL, low familial likelihood for autism
$R^2$ /$R^2$ Adjusted = 0.19/0.10
$F(11, 107) = 2.15$, $P = .03$
[a]Reference category: Graduate degree

© 2023 St. John T et al. *JAMA Network Open.*

eTable 9. A-not-B Total Score by Parietal Lobe, Sex and Likelihood Group Model Fit Results (with Maternal Education)

| | Estimate (95% CI) | SE | *P*-value | $\eta^2_p$ |
|---|---|---|---|---|
| Intercept | 33.40 (7.73, 59.07) | 12.95 | .01 | 0.06 |
| Mullen Early Learning Composite | 0.28 (0.06, 0.49) | 0.11 | .01 | 0.06 |
| Maternal Education (High School)[a] | 2.96 (-6.50, 12.42) | 4.77 | .54 | 0.00 |
| Maternal Education (College)[a] | 0.74 (-7.81, 9.30) | 4.32 | .86 | 0.00 |
| Sex (Female) | 10.70 ( -1.94, 23.35) | 6.38 | .10 | 0.03 |
| Likelihood Group (HL) | -5.97 (-16.16, 4.22) | 5.14 | .25 | 0.01 |
| Total Cerebrum | -8.06 (-18.86, 2.75) | 5.45 | .14 | 0.02 |
| Total Parietal Lobe | 6.34 (-6.38, 19.07) | 6.42 | .33 | 0.01 |
| Sex (Female):Likelihood Group (HL) | -11.08 (-26.11, 3.96) | 7.58 | .15 | 0.02 |
| Sex (Female):Total Parietal Lobe | 14.92 (1.84, 28.01) | 6.60 | .03 | 0.05 |
| Likelihood Group (HL):Total Parietal Lobe | 3.70 (-6.57, 13.98) | 5.18 | .48 | 0.01 |
| Sex (Female):Likelihood Group (HL):Total Parietal Lobe | -17.79 (-33.36, -2.22) | 7.86 | .03 | 0.05 |

Abbreviations: HL, high familial likelihood for autism; LL, low familial likelihood for autism
$R^2$ /$R^2$ Adjusted = 0.19/0.11
$F(11, 106)$= 2.28, $P$ = .02
[a]Reference category: Graduate degree

© 2023 St. John T et al. *JAMA Network Open.*

eTable 10. A-not-B Total Score by Occipital Lobe, Sex and Likelihood Group Model Fit Results (with Maternal Education)

| | Estimate (95% CI) | SE | *P*-value | $\eta^2_p$ |
|---|---|---|---|---|
| Intercept | 35.36 (8.55, 62.17) | 13.52 | .01 | 0.06 |
| Mullen Early Learning Composite | 0.24 (0.02, 0.47) | 0.11 | .04 | 0.04 |
| Maternal Education (High School) | 5.76 (-4.17, 15.68) | 5.00 | .25 | 0.01 |
| Maternal Education (College) | 1.80 (-7.16, 10.76) | 4.52 | .69 | 0.00 |
| Sex (Female) | 5.66 (-7.78, 19.09) | 6.78 | .41 | 0.01 |
| Likelihood Group (HL) | -6.39 (-17.62, 4.84) | 5.66 | .26 | 0.01 |
| Total Cerebrum | 3.03 (-3.14, 9.21) | 3.11 | .33 | 0.01 |
| Total Occipital Lobe | -4.60 (-16.33, 7.14) | 5.92 | .44 | 0.01 |
| Sex (Female):Likelihood Group (HL) | -4.57 (-20.41, 11.27) | 7.99 | .57 | 0.00 |
| Sex (Female):Total Occipital Lobe | 3.86 (-9.99, 17.72) | 6.99 | .58 | 0.00 |
| Likelihood Group (HL):Total Occipital Lobe | 5.00 (-6.75, 16.76) | 5.93 | .40 | 0.01 |
| Sex (Female);Likelihood Group (HL):Total Occipital Lobe | -7.88 (-24.33, 8.57) | 8.30 | .34 | 0.01 |

Abbreviations: HL, high familial likelihood for autism; LL, low familial likelihood for autism
$R^2$ /$R^2$ Adjusted = 0.13/0.03
$F(11, 105) = 1.37$, $P = .20$

© 2023 St. John T et al. *JAMA Network Open.*

eReferences

1. Lord, C., Rutter, M., DiLavore, P. C., Risi, S., Gotham, K., Bishop SL. *Autism Diagnostic Observation Schedule, 2nd Edn. (ADOS-2) Manual (Part I): Modules 1-4*. Western Psychological Services; 2012.
2. Lord C, Rutter M, Le Couteur A. Autism Diagnostic Interview-Revised: A revised version of a diagnostic interview for caregivers of individuals with possible pervasive developmental disorders. *J Autism Dev Disord*. 1994;24(5):659-685. doi:10.1007/BF02172145
3. Avants BB, Tustison NJ, Song G, Cook PA, Klein A, Gee JC. A reproducible evaluation of ANTs similarity metric performance in brain image registration. *Neuroimage*. 2011;54(3):2033-2044. doi:10.1016/j.neuroimage.2010.09.025
4. Kim SH, Fonov VS, Dietrich C, et al. Adaptive prior probability and spatial temporal intensity change estimation for segmentation of the one-year-old human brain. *J Neurosci Methods*. 2013;212(1):43-55. doi:https://doi.org/10.1016/j.jneumeth.2012.09.018

© 2023 St. John T et al. *JAMA Network Open.*
