# Supplementary material for: Association of Sex With Neurobehavioral Markers of Executive Function in 2-Year-Olds at High and Low Likelihood of Autism
Source: JAMA Netw Open. 2023 May 4;6(5):e2311543. doi: 10.1001/jamanetworkopen.2023.11543 (PMC10160873; doi:10.1001/jamanetworkopen.2023.11543)
Supplement: Supplement 2. — Nonauthor Collaborators [file jamanetwopen-e2311543-s002.pdf]

Supplemental Online Content: Nonauthor Collaborators
*First name, last name, and suffix (if applicable) are required and will appear in PubMed.

***Group Name(s): IBIS Network**

***First Name and Middle Initial(s)**	***Last Name**	***Suffix (eg, Jr, III)**	Academic Degrees	Institution	Location (city, state/province, country)	Role or Contribution, eg, chair, principal investigator	Group (if more than 1 Group listed in the byline) and/or Subgroup (eg, Steering Committee)
Jed T.	Elison		PhD	University of Minnesota	Minneapolis, MN, USA		
Jason J.	Wolff		PhD	University of Minnesota	Minneapolis, MN, USA		
Mark D.	Shen		PhD	University of North Carolina	Chapel Hill, NC, USA		
Jessica B.	Girault		PhD	University of North Carolina	Chapel Hill, NC, USA		
J. Chad	Chappell		MA	University of North Carolina	Chapel Hill, NC, USA		
Kinh	Truong		PhD	University of North Carolina	Chapel Hill, NC, USA		
Dennis	Shaw		MD	University of Washington	Seattle, WA, USA		
Kelly N.	Botteron		MD	Washington University School of Medicine in St. Louis	St. Louis, MO, USA		
Robert C.	McKinstry		MD	Washington University School of Medicine in St. Louis	St. Louis, MO, USA		
John N.	Constantino		MD	Washington University School of Medicine in St. Louis	St. Louis, MO, USA		
John R.	Pruett	Jr.	MD	Washington University School of Medicine in St. Louis	St. Louis, MO, USA		
Meghan R.	Swanson		PhD	University of Texas at Dallas	Richardsoon, TX, USA		
Guido	Gerig		PhD	New York University	New York, NY, USA		
Alan C.	Evans		PhD	Montreal Neurological Institute	Montreal, Quebec, Canada		
Leigh C.	MacIntyre		BSc	Montreal Neurological Institute	Montreal, Quebec, Canada		
Samir	Das		BSc	Montreal Neurological Institute	Montreal, Quebec, Canada		
D. Louis	Collins		PhD	Montreal Neurological Institute	Montreal, Quebec, Canada		
Vladimir	Fonov		PhD	Montreal Neurological Institute	Montreal, Quebec, Canada		
