# Supplementary material for: Association of Sex With Neurobehavioral Markers of Executive Function in 2-Year-Olds at High and Low Likelihood of Autism
Source: JAMA Netw Open. 2023 May 4;6(5):e2311543. doi: 10.1001/jamanetworkopen.2023.11543 (PMC10160873; doi:10.1001/jamanetworkopen.2023.11543)
Supplement: Supplement 3. — Data Sharing Statement [file jamanetwopen-e2311543-s003.pdf]

Data Sharing Statement

St. John. Association of Sex With Neurobehavioral Markers of Executive Function in 2-Year-Olds at High and Low Likelihood of Autism. *JAMA Netw Open*. Published May 04, 2023. doi:10.1001/jamanetworkopen.2023.11543

Data
Data available: No
